# The Immunomodulatory Properties of β-2,6 Fructans: A Comprehensive Review

**DOI:** 10.3390/nu13041309

**Published:** 2021-04-15

**Authors:** Ian D. Young, Dimitrios Latousakis, Nathalie Juge

**Affiliations:** 1 Quadram Institute Bioscience, Norwich Research Park, Norwich NR4 7UQ, UK; dimitris.latousakis@quadram.ac.uk; 2 Universitätsklinik für Viszerale Chirurgie und Medizin, Inselspital, Bern University Hospital, Department for BioMedical Research (DBMR), University of Bern, Murtenstrasse 35, 3008 Bern, Switzerland

**Keywords:** fructan, levan, immunomodulatory, microbiota, gut health, immunity, fructose, polysaccharide, fructooligosaccharide, exopolysaccharide

## Abstract

Polysaccharides such as β-2,1-linked fructans including inulin or fructose oligosaccharides are well-known prebiotics with recognised immunomodulatory properties. In recent years, other fructan types covering β-2,6-linked fructans, particularly microbial levans, have gained increasing interest in the field. β-2,6-linked fructans of different degrees of polymerisation can be synthesised by plants or microbes including those that reside in the gastrointestinal tract. Accumulating evidence suggests a role for these β-2,6 fructans in modulating immune function. Here, we provide an overview of the sources and structures of β-2,6 fructans from plants and microbes and describe their ability to modulate immune function in vitro and in vivo along with the suggested mechanisms underpinning their immunomodulatory properties. Further, we discuss the limitations and perspectives pertinent to current studies and the potential applications of β-2,6 fructans including in gut health.

## 1. Introduction

Carbohydrates such as oligosaccharides and polysaccharides (PS) are one of the most abundant compounds on earth comprising >50% of the world’s biomass [1]. These diverse structures are derived from plants, microorganisms and mammals and synthesised by a vast range of enzymes [1,2,3]. PS and oligosaccharides fulfil many critical functions in living organisms including sustenance, storage reserves and as structural support [1,3,4,5,6,7,8]. PS are homopolymers or heteropolymers that are typically made up of >20 sugar monomers [1]. The large diversity of PS reflects the range of natural monomeric sugars (e.g., d-glucose, d-mannose, d-fructose, d-xylose, d-galactose, d-fucose, d-glucuronic acid, l-rhamnose, among many others) and pyranose and furanose ring formations that can make up countless assortments of di-, tri- and oligosaccharides and PS of wide ranging molecular weights [1,3]. These structures can be linked by α-or β-glycosidic bonds and may adopt many variations of branching combinations from simple structures to highly complex macromolecules [1,3]. 

PS from plants, as well as from microbes, can be consumed as part of the human diet [9,10,11,12] For example, starch can be broken down by host-derived enzymes to glucose units for energy [13]. Alternatively, nondigestible PS (NDP) are primary components of dietary fibres [12,14] and are resistant to human gut enzymes in the upper part of the gastrointestinal (GI) tract [15,16]. These NDP are fermented by resident commensal microbes in the large intestine [15,16]. Consumption of dietary fibres by humans has been linked to several health and physiological benefits [15,17]. For example, a reduced risk of death and mortality from diabetes, cancer, infections, and respiratory disease was shown by meta-analysis to be associated with increased whole grain or cereal fibre consumption [15,18], and diets rich in dietary fibres are linked to decreased blood insulin and glucose levels, reduced food transit time, increased satiety, weight loss promotion, cholesterol-lowering effects, improved mineral absorption and reduced blood pressure, among other effects [13,15]. Dietary β-glucans and fructans are well-known examples of dietary fibres that contribute to some of these health benefits [19,20]. 

Microbial PS synthesised from pathogenic or commensal microbes comprise a vast and complex array of structures [21,22,23] with many attached to other macromolecules to form glycoconjugates such as glycolipids and glycoproteins [1,3,6,7]. Microbial PS can be found in the cytoplasm as storage PS, or associated with the cell wall [24] including as capsular PS on the outer surface [21,23] and lipopolysaccharides (LPS) anchored to the cell membrane [24,25]. They are also found as exopolysaccharides (EPS) secreted into the extracellular environment or loosely associated with the bacterial cell surface [1,24]. PS from microbes, for example those inhabiting the human body such as the gut [26] play biological roles ranging from biofilm formation to immunomodulators. For example, EPS are a component of biofilms aiding in their function, stability and maintenance [27,28]. EPS can interact with each other and form the matrix that encompasses microbial cells [28]. Biofilms have mainly be studied in pathogens [29]. Among commensal or probiotic bacteria, *L. johnsonii* EPS has been shown to modulate biofilm formation in vitro [30], and *B. subtilis* EPS has been reported to improve the stability of floating biofilms [31]. Further, it has been suggested that biofilms formed by EPS-producing probiotic bacteria may aid host protection to injury or pathogenic insults [24]. Many microbial PS are also known to influence host immune function [1,21,24,32,33,34,35]. For example, EPS-producing strains of Bifidobacteria showed a reduced ability to induce host immune responses in vivo compared to EPS knock-out strains, supporting the role of EPS from commensal bacteria in maintaining host-microbial mutualism [32]. Microbial or plant PS have also been shown to modulate cytokine production by immune cells in vitro [24,36,37,38,39].

Fructans represent an important class of PS in plants and microbes, in terms of occurrence and biological function. Poly- or oligosaccharide β-2,1 inulin-type fructans typically contain only β-2,1-linked fructose residues ranging up to 60 monomeric units [40] and are well-known prebiotics with recognised immunomodulatory properties as extensively reviewed [41,42,43]. Another type of fructans, containing β-2,6-linked fructose residues, are produced by microbes where they are named levan, and in plants, herein termed plant β-2,6 fructans.

**Figure 1:**
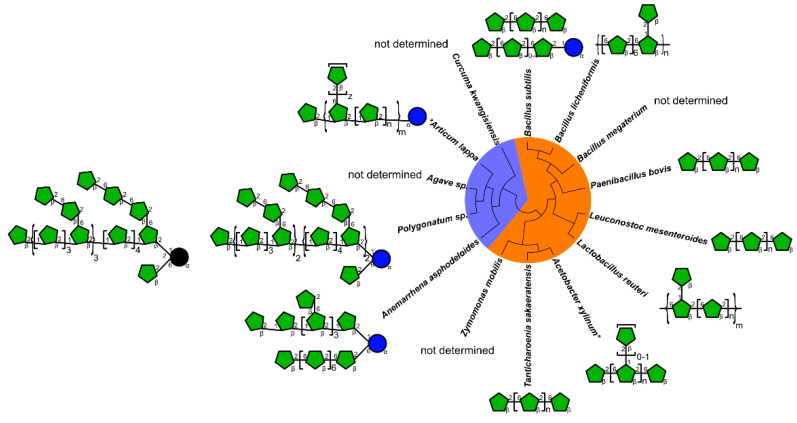
Schematic structural representation of plant or microbial β-2,6 fructans with reported immunomodulatory properties. Microbial levan or plant β-2,6 fructans are shaded orange or blue, respectively. Green pentagons represent fructose, blue circles, glucose and black circles, *O*-acetylated glucose. * Tentative structure. Also see Table 1 and Table 2 for descriptive structures.

Here, we will provide an overview of plant and microbial β-2,6 fructans of different structures, focusing on their in vitro and in vivo immunomodulatory properties (Table 1, Figure 1). We will also discuss the suggested mechanisms underpinning their immunomodulatory function while outlining the limitations and perspectives pertinent to the field, as well as the potential applications of β-2,6 fructans including in gut health.

## 2. Overview of β-2,6 Fructans: Occurrence, Synthesis and Function

### 2.1. Plant β-2,6 Fructans

Plants utilise fructosyltransferases to synthesise fructans by adding fructose residues on to sucrose leading to the production of different fructofuranose-linked structures [44,45,46,47,48]. Plant fructans (β-2,6 fructans or β-2,1 inulin-type fructans) are polymers of fructose found as storage PS in ~15% of higher plants such as forage grasses, cereals and vegetables [49]. Ιnulin, known as a common dietary fibre and prebiotic [17], has received much attention for its immunomodulatory properties including the ability to induce beneficial effects via the gut microbiota or through direct interactions with the immune system [41,43,46,50,51], however, these effects are less known in plant fructans containing β-2,6 linkages. 

Plant β-2,6 fructans include plant levans, also named phleins, which are fructans that generally contain linear β-2,6-linked fructose chains and are found in a small number of plant species like grasses [52,53]. In addition, mixed linkage plant fructans can contain β-2,6-fructose-linked residues with β-2,1-branching which are typically termed graminians [52]. Plant graminians also can comprise more complex structures involving additional fructose-linked branching or as neoseries structures [52]. Further, some plant fructans contain a β-2,1-backbone with β-2,6 linkages, for example [54]. β-2,6 fructans can be produced by some plant species including *Agropyron cristatum* [55], *Pachysandra terminalis* [56] and *Curcuma kwangsiensis* [57], among others [44,48]. These plant β-2,6 fructans are nonstructural storage carbohydrates located in leaf and stem sheaths and are generally low in molecular weight (Mw) [48]. In addition, this review includes plant inulin-type fructans with β-2,6 branching points, for example ALP-1 produced by *Arctium lappa* (discussed in Section 3 and Section 4; see Table 1 for structure). 

### 2.2. Microbial Levan

Microbial levan is typically a large β-2,6 fructofuranose polymer, a β-2,6 fructan [10], that is linear or can contain β-2,1 branching [48,58,59]. Many bacteria are capable of synthesising levan including gut commensal *Lactobacillus reuteri* [60], and *Streptococcus mutans* or *S. salivarius* found in the oral cavity [61,62], as well as *Bacillus subtilis* [9,63], *B. amyloliquefaciens* [64], *L. citreum* [65], *Zymomonas mobilis* [66], *Pseudomonas syringae pv* [67], *Erwinia herbicola* [68], *Microbacterium laevaniformans* and *Serratia levanicum* [48,69]. In microbes, levan is synthesised by levansucrase (E.C 2.4.1.10), a fructosyltransferase belonging to family 68 of glycoside hydrolases (GH68) according to the CAZy (carbohydrate-active enzymes) database (www.cazy.org, accessed on 13 April 2021) [48]. Generally, levansucrases are secreted into the extracellular environment but can also be attached to the bacterial cell surface [48,58]. Levansucrase binds to a substrate, such as sucrose, and adds fructose molecules to a growing fructose chain [48,58]. Typically, this process produces a glucose-capped β-2,6-linked fructofuranose polymer containing no or some β-2,1 branching [48]. 

Levan can be produced by bacterial fermentation or in vitro using recombinant levansucrase heterologously made in *Escherichia coli* [48,70,71,72]. In addition, yeast such as *S. cerevisiae* has been used for levan production, although, this is less common [48,73]. The degree of branching (up to 13% branching has been reported [48]) and molecular weight of microbial levan depend on the microbial source and the production method [48,59]. In addition, low-branched microbial levan has been shown to be produced by bacteria such as *S. levanicum* [69]. Moreover, some species of acetic acid bacteria produce linear levan [74]. In general, microbial levans form high molecular weight polymers [48] such as levan produced by *Serratia* sp. which can reach up to 4,400,000 Da [48,69]. However, some bacteria have been shown to produce both low and high molecular weight levans, for example from *B. subtilis* natto [48,75]. The conditions known to affect the molecular weight of microbial levan during its production include temperature and levansucrase concentration [48]. 

Microbial levan is an amphiphilic molecule known to adopt a spherical conformation in aqueous solution, and, therefore is commonly referred to as a nanoparticle [48,58,59,72]. Microbial levan has been proposed for its use in several industries including in aquaculture [48,76] and as packaging/films [48]. Further, levan has been incorporated in products from the food industry, for example in dairy products or bread [48,77,78]. It is also present in fermented food such as natto (fermented soybean) [9,10]. Microbial levan may also be relevant to the pharmaceutical industry, for example, as a nanoparticle for delivering drugs including antibiotics [48,79]. 

In terms of its biological role, microbial levan acts as a constituent of biofilm matrices in some bacteria [31,80] and has also been suggested to contribute towards plant pathogen fitness and virulence [48,81,82]. Further, in soil-resident bacteria, levan has been shown to aid in tolerance to salt and desiccation as well formation of cell aggregates on abiotic surfaces suggesting a role for levan in environment adaptions of bacteria under high osmotic stress and in biofilm formation [80]. In addition, levan has been suggested to act as a nutritional reservoir [31] perhaps as an energy source under conditions like starvation [48]. Further, levan has been suggested to promote colonisation of bacteria in the gut [60] and to act as a prebiotic in vitro [71,83,84,85,86,87] although in vivo evidence is scarce [88,89]. The human gut symbiont *B. thetaiotaomicron* can utilise levan [90], supporting a potential role of levan as a prebiotic in humans. In addition, isolates of levan-producing strains such as *B. subtilis* sp isolates or *L. reuteri* LTH5794 have been detected in the faeces of healthy humans [91,92,93,94], yet further work is warranted to verify that levan is produced in the gut by resident commensal bacteria in the GI tract. In addition, microbial levan has been suggested to elicit bioactivity or confer several health benefits including anticancer/antitumour [66], antipathogenic [78], antidiabetic [95], cholesterol-lowering [48,96], antioxidant [97,98], antiviral [99] or antiobesity properties [100].

The next sections provide an overview of the in vitro (Table 1) and in vivo (Table 2) evidence for the immunomodulatory properties of β-2,6 fructans from plants and microbes.

## 3. The Immunomodulatory Properties of Microbial Levan and Plant β-2,6 Fructans In Vitro

### 3.1. Effect of β-2,6 Fructans on Cytokine or Chemokine Production and Immunity

Microbial levan and plant β-2,6 fructans have been shown to modulate cytokine production by immune cells in vitro, as summarised in Table 1, which includes structural information. For example, the soil-bacterium *B. licheniformis* produces a high-molecular weight (Mw) levan EPS (2,000,000 Da) containing β-2,1 branching which induced the production of proinflammatory cytokines IL-6 and TNF-α by human whole blood cells [101]. Another high Mw (>2,600,000 Da) but linear levan isolated from *Paenibacillus* sp. nov BD3526 induced TNF-α production by isolated murine splenocytes but not IL-2, IL-4, IL-6, IL-10, IL17A or IFN-γ suggesting a marginal inflammatory effect [102]. The low Mw microbial levans produced by *B. subtilis* natto CCT7712, a β-2,6 fructooligosaccharide mix, increased expression of TNF-α and proinflammatory chemokine IL-8 in human-derived ovarian carcinoma cells [104]. Although these high and low Mw microbial levans both induce TNF-α, the discrepancy with other cytokines may be partly explained by the structural differences of levan such as branching/linearity and Mw, and/or the different cell types used in the assays. The mechanisms underpinning the proinflammatory immunomodulatory properties of microbial levan remain poorly understood. However, a study by Xu and colleagues (2006) showed that the production of proinflammatory cytokines TNF-α and IL-12 p40 by *B. subtilis* natto levan in monocyte/macrophage cell lines [10], was not due to bacterial LPS, a potent TLR4 ligand, as (i) levan was sourced from Gram-positive bacteria and (ii) IL-12 p40 production was not dampened using the LPS inhibitor, polymyxin B [10] (LPS contamination is further discussed in Section 5). *B. subtilis* natto levan induced TNF-α and IL-12 p40 production in murine primary peritoneal and spleen cells. TNF-α production in peritoneal cells was strongly dependent on TLR4, and reduced in TLR2 KO cells [10]. In addition, microbial levan treated with LPS-inhibitor polymyxin-B activated human TLR4-transfected HEK293 cell lines at low concentrations, supporting that cytokine production by levan was TLR-mediated. In addition, some of the microbial levans tested were shown to induce anti-inflammatory cytokines in vitro. For example, levan from *L. mesenteroides* S81 found in sourdough did not induce cytokines TNF-α, IL-12 or IL-10 in HT-29 cells (human epithelial cell line [114]) but did stimulate IL-4 production [98]. IL-4 is an anti-inflammatory cytokine that plays a key role in the type 2 inflammatory response and allergy [115]. Furthermore, treatment of LPS-challenged RAW 264.7 macrophages with ALP-1 (see Table 1 for structure) stimulated anti-inflammatory cytokine IL-10 and reduced proinflammatory cytokines TNF-α, IL-6 and IL-1β in a dose-dependent fashion [54].

Similar variations in cytokine production are observed for plant β-2,6 fructans (Table 1). *Anemarrhena asphodeloides*, a plant commonly found in eastern Asia and used as a traditional medicine in China, produces a low Mw neokestose β-2,6 fructan termed AAP70-1 which was shown to induce the production of IL-6, IL-1β and TNF-α in RAW 264.7 macrophages [110]. Moreover, *Polygonatum odoratum* and *P. cyrtonema*, plant species of the *Asparagaceae* family produces neokestose fructans with β-2,1-linked linear chains (instead of β-2,6 backbone) and β-2,6 branching, POP-1 and PCP-1, respectively. POP-1 and PCP-1 induced IL-6 production by RAW264.7 macrophages with PCP-1 exhibiting a higher potency [111]. The authors suggested that the acetyl group on the glucose residue of PCP-1 fructan may be responsible for the difference in IL-6 production, as the immunomodulatory properties of other acetylated PS exhibited stronger immunomodulatory activity than nonacetylated PS [111,116]. This supports the notion that different levan structures may explain in part some of the discrepancies observed in cytokine production in vitro. However, branching structures are only seldom reported (Table 1) and more thorough and systematic carbohydrate analyses such as GC-MS linkage analysis would help advance our understanding of structure-activity of levans, as shown in other studies [57,60]. The mechanisms underpinning the immunomodulatory properties of plant β-2,6 fructans remain to be investigated. Agave fructans isolated from *Agave salmiana* comprising highly branched fructose polymers with both β-2,6 and β-2,1 linkages [112] appeared to directly induce the expression of T-cell-associated transcription factors FOXP3 and Tbet in peripheral blood mononuclear cells [113]. 

Using well-characterised high-purity microbial levans and plant β-2,6 fructans to assay immune modulation across multiple cell types as well as in vivo (Section 4) is required to address the discrepancies between the effect of levans on cytokine modulation.

### 3.2. Effect of Levan on Macrophage NO Production, Phagocytic Activity and Cell Proliferation

Associated with their ability to influence immune cell responses, microbial levan and plant β-2,6 fructans have been reported to modulate nitric oxide (NO) production, a well-known immunomodulatory product of activated macrophages [117], as well as affect macrophage phagocytosis (see Table 1 for structural details when available). For example, plant β-2,6 fructan ALP-1 showed an anti-inflammatory effect by decreasing LPS-induced NO production in RAW 264.7 macrophages [54] while AAP70-1 showed no induction of NO [110]. Plant AAP70-1, POP-1 and PCP-1 also showed an ability to enhance the phagocytic activity of RAW 264.7 macrophages, and PCP-1 stimulated macrophage cell proliferation at high concentrations (>200 μg/mL) [111]. In line with these findings, a low Mw branched β-2,6 fructan with a β-2,6-linked main chain from the plant species *Curcuma kwangsiensis* also increased the phagocytic activity of RAW264.7 macrophages (by 39%), while LPS resulted in an 82% increase and induced macrophage proliferation [57]. The levan soil bacterium *Tanticharoenia sakaeratensis,* which produces a high Mw levan EPS, was also shown to induce NO production in RAW264.7 macrophages in a dose-dependent manner and the use of the LPS-inhibitor polymyxin B confirmed that the effects were not attributable to LPS contamination [105]. Similarly, both *Z. mobilis* levan and di-D-fructose-2,6′:6,2′-dianhydride (DFA-IV)-a levan derivative disaccharide consisting of 2 fructose residues enzymatically produced using levan fructosyl transferase [106] were found to increase NO production as well as NO synthase (iNOS) expression [108]. Other microbial levans have been reported to induce immune cell proliferation. This is for example the case for levans from *B. licheniformis* 8-37-0-1 and *Paenibacillus bovis* sp. nov BD3526 which were shown to induce the proliferation of murine splenocytes [102,103]. However, it is not known whether spleen cell proliferation by microbial levan or plant β-2,6 fructans includes macrophage proliferation, and what is the biological impact of the proliferative potential of microbial levan or plant β-2,6 fructans on immune cells.

Overall, microbial levan as well as plant β-2,6 fructans were shown to increase phagocytic activity of macrophages, which may be applied to promote animal health or reduce infection (as described in Section 4). Further, the contrasting findings of microbial levans (and levan derivative DFA-IV) or plant β-2,6 fructans on NO production is unclear but may be related to their purity, Mw or compositional structure. Further studies are warranted to compare how different β-2,6 fructan structures from plant and microbial sources may affect macrophage function which could be used for beneficial applications.

### 3.3. Effect of Microbial Levan on Gut Barrier Function

Intestinal epithelial cells are being increasingly recognised for their role in immune function and are important for providing protection to the host against microbial invaders [118,119]. Levan nanoparticles produced in vitro using recombinant levansucrase Lsc3 from *P. syringae pv*. Tomato, were tested for their effect on epithelium integrity using human Caco-2 cells grown on transwells by transepithelial electrical resistance (TEER) [72]. While sodium dodecyl sulfate (SDS) increased membrane permeability, cells treated with levan nanoparticles showed a dose-dependent moderate decrease in intestinal permeability compared to medium controls, supporting a possible role of levan in strengthening the gut barrier [72]. In another study, pretreatment of IPEC-J2 cells (porcine enterocyte model [120]) with β-2,6-linked fructose disaccharide DFA-IV led to decreased membrane permeability post LPS-challenge, determined by TEER and fluorescein isothiocyanate (FITC) dextran measurements [121]. The authors also showed that DFA-IV was associated with intestinal wound healing properties in vitro and in vivo through measuring specific genes associated with differentiation, proliferation and cell migration [121]. Together, these studies indicate that levan shows potential to improve intestinal barrier function in vitro or associated wound healing in the intestine, although due to the limited amount of studies and different methodologies between studies, further work is required to confirm these in vitro findings as well as test the effect of levan on intestinal barrier function in vivo.

IgA, the most abundant immunoglobulin (Ig) in animals, is secreted primarily in mucosal tissues such as the intestine [122]. IgA plays a key role in gut barrier function, protecting the host by neutralising pathogenic threats such as viruses, halting bacterial contact to the intestinal epithelium, and facilitating the clearance of large biomolecules, among other functions [122]. *L. mesenteroides* NTM048 levansucrase-synthesised levan and *L. mesenteroides* NTM048 EPS but not *B. subtilis* levan were shown to induce IgA production in isolated murine peyer’s patches [109]. These differences may be due to the capacity of IgA to target different microbial antigens [123]. For example, IgA was proposed to target proteins associated with the *B. thetaiotaomicron* fructan-associated PS utilization locus [123]. Therefore, this mechanism may play an indirect role in gut colonisation and host-microbial mutualism [123]. However, further work is needed to assess the role of microbial levan (or its derivative DFA-IV) from different sources on IgA induction, and whether this can be used as a route to modulate beneficial members of the gut microbiota. 

## 4. The Immunomodulatory Properties of Microbial and Plant β-2,6-Associated Fructans In Vivo

There are limited studies investigating the impact of β-2,6 fructans on human health [124,125], and to the best of our knowledge, there have been no reports investigating the immunomodulatory properties of plant β-2,6 fructans or microbial levans in humans. Below we discuss studies that reported the immunomodulatory properties of microbial levan and plant β-2,6 fructans in vivo which have mostly been demonstrated using murine or fish models (Table 2).

### 4.1. Effect of β-2,6 Fructans on Immune Responses in Mice

The first studies investigating microbial levan and immunity-associated modulations in mice date back as far as 1948, with levan from *S. salivarius* on MUMPs virus multiplication [126]. Levan from *Aerobacter laevenicum* was later shown to induce levan-specific antibody responses which may also be cross-reactive with the β-2,1 fructose polymer inulin [127,128]. More recent evidence for immune modulation by microbial levan or β-2,6 fructans in mice is limited to studies using microbial levans from *L. reuteri* and *B. subtilis* natto and plant fructan ALP-1.

In vivo, *L. reuteri* 100-23, which produces a relatively low Mw branched levan (see Table 1 for structure), was found to modulate T cell responses in the spleen of rats, as splenic FOXP3+ CD4+ regulatory T cells were higher in rats colonised with the wild-type strain as compared to a fructosyltransferase (ftf) KO *L. reuteri* strain incapable of producing EPS levan [60]. This study indicates that levan produced in situ may play a role in the evolutionary adaptation of *L. reuteri* to a sucrose-rich gut environment by modulation of the immune system [60]. Microbial levan has also been suggested to modulate Th2 helper cell responses in allergy models. Here, *B. subtilis* natto levan orally-administered to mice decreased levels of ovalbumin-(OVA)-induced serum IgE while there was no difference seen with IgG2a or IgG1 [10]. IgE as well as IL-4 are involved in Th2 responses while IgG2a for example is associated with Th1 responses [10]. Following immunisation with OVA, *B. subtilis* natto levan decreased IL-4 levels in splenic T cells isolated from mice, but there was no difference in IFN-γ levels, suggesting that levan suppressed the Th2 response [10]. Interestingly, an older study by Bartocci and colleagues (1982) reported that levan from *Aerobacter levanicum* elevated the delayed-type hypersensitivity response in mice, also known as a type IV hypersensitivity reaction: a type of cell-mediated allergic immune reaction [129,130]. These studies, although limited to discrete microbial levans, provide some mechanistic insights into the immunomodulatory properties of levan reported in vitro, particularly through the modulation of T cell responses and interaction with TLR4 (see Section 3.1). 

Dietary ALP-1 led to increased IL-10 in the serum and colon of dextran sodium sulfate (DSS)-induced mice, while proinflammatory cytokines TNF-α, IL1β and IL-6 were decreased, and IgA levels also increased in the colon [131]. In addition, ALP-1 intake appeared to alleviate the damaging effects to the colon induced by colitis [131]. ALP-1 was also investigated in a separate study by Zhang and colleagues (2019) using mice challenged with LPS [54]. Levels of serum proteins TNF-α, IL-1β, IL-6 were significantly decreased in the LPS-challenged mice receiving ALP-1 supplementation whereas IL-10 was further elevated [54]. These studies suggest that the anti-inflammatory properties of levan are associated with the host physiological status, which may explain some of the discrepancies with and limitations of the in vitro assays which, for the vast majority, were carried out in nonchallenged conditions e.g. without LPS pretreatment (see Section 3.1).

Lastly, a recent study by Ragab and colleagues (2020) investigated the effect of levan from *B. subtilis* isolates associated with honey (see Table 2 for structure) on peptic ulcers in rats [132]. This low Mw levan induced ulcer alleviation, as well as decreased NF-κB levels but had no antimicrobial effect in vitro on *Helicobacter pylori*, a pathogen associated with producing gastric ulcers [133], suggesting that the mechanism was due to levan’s prebiotic and/or anti-inflammatory properties [132]. These effects are in accordance with previous reports showing that DFA-IV could improve intestinal wound healing in vivo [121], again supporting the view that the anti-inflammatory effects of levans may only be tractable in a disease model. 

### 4.2. Effect of β-2,6 Fructans on the Immune Response in Fish

There have been several studies reporting microbial levan as immunomodulators or prebiotic agents in aquaculture [76] by modulating cytokine production and/or conferring improved resistance to pathogenic or chemical insults [76,134,135,136]. For example, fish (*Cyprinus carpio* fry) fed levan derived from *Bacillus megaterium* that were exposed to low doses of the insecticide Fipronil to induce stress responses, showed elevated serum globulin, total protein and lysosome activity, and increased white blood cell (WBC) counts, which are likely associated with increased phagocytic activity [135]. Moreover, Gupta and colleagues (2018) found that high Mw (750,000 Da) branched levan derived from *Acetobacter xylinum* NCIM 2526 induced TNF-α, IL-1β, and IL-12p40 expression in several organs and reduced the expression of IL-10 in the intestines of *Labeo rohita* fingerlings after challenge with *Aeromonas hydrophila*, a common pathogenic bacteria in fish [137]. Dietary levan supplementation after pathogenic challenge also increased lysozyme activity and the respiratory burst (release of reactive oxygen species) in serum and blood. Gupta and colleagues (2020) then showed that *A. xylinum* levan fed to *A. hydrophila*-challenged *L. rohita* fingerlings, led to increased Ig levels [138]. This levan also upregulated TLR22 expression (a PRR exclusive to fish) and IFN-γ in several organs of the fish including the intestine while the expression of TGF-β was mostly reduced [138]. 

Taken together, studies in fish exposed to insults showed multiple immunomodulatory properties across several studies. Microbial levan may elevate the immune response to aid in response to chemical or pathogenic challenge such as inducing or lowering proinflammatory cytokine induction, respectively, or increasing TLR expression or Ig responses, all contributing to mounting an appropriate immune response, that may be beneficial for aquaculture.

### 4.3. Indirect Effects of β-2,6 Fructan-Induced Immune Responses

An inherent factor influencing the immunomodulatory properties of fructans in vivo may be through the modulation of the gut microbiota, as demonstrated for β-2,1 fructans [43]. However, little is known on the indirect immunomodulatory effects of microbial levan or plant β-2,6 fructans via the microbiota in vivo and most studies are based on models of pathogenic or inflammatory challenge. ALP-1 supplementation was shown to modulate gut microbiota composition, including a slight increase in beneficial *Lactobacillus* genus in the colon of DSS-induced mice compared to control [131]. In LPS-challenged mice, ALP-1 appeared to increase Proteobactera and Firmicutes. At the genus level, ALP- 1 led to an increase of *Lactobacillus* and *Odoribacter* and decreased *Bacteroides* in the LPS-induced mice [54]. In line with these changes in microbial profiles, ALP-1 treatment in LPS-challenged mice also increased faecal short chain fatty acids (SCFA). Together, these studies suggest that the observed anti-inflammatory effects of ALP-1 in diseased models may be attributed to its prebiotic properties through a modulation of the gut microbiota.

In pigs, Li and Kim (2013) investigated changes in microbiota composition, and the prebiotic and immunomodulatory activity of a commercial fructan, a *Z. mobilis* levansucrase-derived high Mw levan [139] (See Table 2). Dietary *Z. mobilis* levansucrase-derived levan was shown to increase *Lactobacillus* levels in faeces, indicating a possible prebiotic activity. In addition, prolonged dietary supplementation of this levan to pigs prior to an LPS-challenge modulated immune system factors in blood including increased blood leukocytes, and inhibited serum IL-6 and TNF-α production [139] as also similarly reported using ALP-1 [131], as described above. It is noteworthy that in another study, EPS dextran and levan (from *B. paralicheniformis*; 55,170 Da) was suggested as a potential natural alternative to antibiotics in reducing the growth of intestinal enteric pathogens in broilers [140]. Together, these data suggest that the anti-inflammatory properties of plant and microbial levans in diseased models may be via the modulation of the gut microbiota.

## 5. Conclusions and Perspectives

This review gathered in vitro and in vivo evidence for the immunomodulatory properties of microbial and plant β-2,6 fructans. The large heterogeneity of experimental approaches and in vitro and in vivo models used in these studies, as well as the large range of β-2,6 fructans tested (in terms of structure and biological source) and their degree of purity, render it challenging to attribute distinct effects to specific structures. 

However, despite the variability of the findings, β-2,6 fructans from plants and microbes consistently showed modulation of cell cytokine production in vitro using macrophages, human blood cells, mouse splenocytes, or human epithelial cells. Although the exact mechanisms underpinning the immunomodulatory activities of both microbial and plant β-2,6 fructans as well as other immunomodulatory PS remain elusive, several pathways have been implicated including the modulation of the gut microbiota or their metabolites, or direct interaction with immune cells. One potential underpinning mechanism for microbial levan specifically is through the interaction with TLR4, as proposed for levan from *B. subtilis* natto using TLR4-transfected cells [10], in line with other immunomodulatory PS which have been proposed to interact with PRRs such as TLRs and C-type lectin receptors (CLRs) [10,24,26,36,37,41,141] including β-(2,1) fructans [142,143]. Furthermore, CLRs [144], are expressed in the gut where they play an immunomodulatory role by binding to carbohydrates [145,146,147,148], and may therefore interact with dietary or endogenously produced microbial fructans when accessible to the underlying gut-associated lymphoid tissue (GALT) or systemic circulation. 

When assessing the modulation of immune cell function of microbial levan or plant β-2,6 fructans, or other PS in vitro, it is important to consider their composition and purity [149]. For example, by removing microbe-associated molecular patterns (MAMPs) [26,150,151,152] such as LPS which can induce immunostimulatory effects at very low levels in vitro [150,153]. Current methods include the use of LPS inhibitor polymyxin B [153], Triton-X-114 and alkali-based treatments [152,154,155] but there is a need for more sensitive and efficient methods to remove and more accurately quantify LPS [153,156]. In addition, the huge diversity and complex structural features of plant and microbial PS presents challenges and difficulties when determining their full structures [157,158]. For example, with regards to fructan characterisation, degradation of fructose can occur during hydrolysis-based protocols such as monosaccharide analysis which relies on the use of trifluroracetic acid [3,159] and fructose interconversion to glucose or mannose has been reported to occur under acidic conditions [160]. There is also a need to better characterise the degree of branching which may affect microbial levan or plant β-2,6 fructan bioactivity.

In vivo studies suggest that microbial or plant β-2,6 fructans show anti-inflammatory properties using models of infection or inflammation. However very little work has been done to assess the contribution of the gut microbiota in this process, which is in contrast to the more frequently-studied β-(2,1) fructans for example [43,161,162] and reviewed in [41]. Most in vivo studies investigating the health effects of β-2,6 fructans, particularly microbial levan, reported in this review have been performed in fish or murine experimental models, and studies in humans are lacking. One of the mechanisms supporting the immunomodulatory properties of microbial levan specifically in vivo involves the modulation of regulatory T cell responses in the spleen, but whether this also occurs in GALT remains to be determined. Further work is warranted to determine how β-2,6 fructans from plants or microbes from different sources and of diverse structures and molecular weights may affect cytokine production and gut barrier function in murine models such as DSS-induced colitis in mice where altered permeability may provide facilitated access to GALT as suggested using other PS [163,164]. 

In conclusion, despite the limitations highlighted above, plant and microbial β-2,6 fructans represent a promising group of immunomodulatory PS. Further mechanistic work is warranted to uncover their mode of action using differently-sourced fructans of different molecular weights and structures in well-controlled animal models including if they can directly or indirectly affect the immune system. Human trials combined with metagenomic, transcriptomic, and metabolomic studies will help advance our understanding of how these β-2,6 fructans influence gut health. 

At present, it is too early to select a β-2,6 fructan structure based on plant or microbial sources that could be used in prophylactic or therapeutic treatments of specific conditions although evidence from in vitro and animal studies indicates that microbial levans show great promise as immunomodulatory and/or prebiotic agents, that could be used in the pharmaceutical or food industry, or in animal husbandry. It should be noted that a factor limiting the wide study and application of microbial levan specifically is its low purification yield, high costs or bottlenecks in processing associated with its production on a large scale [165,166]. Plant β-2,6 fructans remain under-studied compared to microbial levan. Further work is required to harness the full potential of microbial levan and/or plant β-2,6 fructans as immunomodulatory and/or prebiotic agents for health and industrial applications.

## Figures and Tables

**Table 1:** Examples of studies describing the immunomodulatory effects of β-2,6 fructans in vitro.

Fructan Origin	Source	Reported Structure	Mw (Da)	Immunomodulatory Effect(s)	Reference
*B. subtilis* natto levan EPS	Microbial	n.d	n.d	↑ TNF-α and IL-12p40 production in monocyte/macrophage cell lines and peritoneal and splenic mouse primary macrophages; TNF-α production in peritoneal cells was TLR4-dependent. The levan also activated TLR4 reporter cells.	[10]
*B. licheniformis* levan EPS	Microbial	β-2,6-linked backbone with β-2,1 branching.	2,000,000	↑ TNF-α and IL-6 production in human whole blood.	[101]
*Paenibacillus* sp. nov BD3526 levan EPS	Microbial	Linear β-2,6 fructofuranose polysaccharide.	>2,600,000	↑ TNF-α production in mouse splenocytes; and ↑ mouse spleen cell proliferation.	[102]
*B. licheniformis* 8-37-0-1 levan EPS	Microbial	β-2,6-linked levan containing β-2,1 branching.	28,260	↑ mouse spleen cell proliferation	[103]
*B. subtilis* natto CCT7712 FOS	Microbial	Mixed DP up to 7: β-2,6-linked fructooligosaccharides (primarily 6-nystose).	n.d	↑ gene expression of IL-8 and TNF-α in human OVCAR-3 cells.	[104]
Levan derived from *L. mesenteroides S81* (found in sourdough)	Microbial	Linear β-2,6 fructan; spherical form in aqueous solution.	n.d	↑ IL-4 in human epithelial model HT-29 cells.	[98]
*Tanticharoenia sakaeratensis* (from soil) levan EPS	Microbial	β-2,6 fructofuranose polymer.	~100,000 to 680,000	↑ NO production in RAW264.7 macrophages	[105]
DFA-IV *and Z. mobilis* levan	Synthetic and Microbial	DFA-IV, disaccharide Di-D-fructose-2,6′:6,2′-dianhydride is a levan derivative disaccharide consisting of 2 fructose residues enzymatically produced using levan fructosyl transferase [106]. *Z. mobilis* levan was n.d but is compositionally described in [107].	DFA-IV, 324 [106]; *Z. mobilis* 6,000,000	Both *Z. mobilis* levan (control) and DFA-IV ↑ phagocytosis of RAW264.7 macrophages; and ↑ iNOS expression and NO production in RAW264.7 macrophages.	[108]
Recombinant levansucrase levan derived from *leuconostoc mesenteroides* NTM048 found in green pea	Recombinant levansucrase	Levan (similar NMR chemical shifts to *B. subtilis* levan).	n.d	↑ IgA in isolated murine ↑ peyer’s patches; however no IgA induction by *B. subtilis* levan.	[109]
Fructan from *Anemarrhena asphodeloides* (AAP70-1)	Plant	β-2,6 fructofuranose linear chain with β-2,1 fructofuranose branching point and terminal α-glucopyranose (neokestose).	2720	↑ IL-6, IL-1β and TNF-α in RAW 264.7 macrophages; ↑ phagocytic function of macrophages.	[110]
Fructans from *Polygonatum odoratum* (POP-1) and *P. cyrtonema* (PCP-1)	Plant	β-2,1 fructofuranose linear chain with β-2,6 side branching and an internal α-glucopyranose (neokestose).	5000	↑ IL-6 and phagocytic activity of RAW264.7 macrophages.	[111]
Fructan from *Arctium lappa* (ALP-1)	Plant	β-2,1 backbone with β-2,6 branching and a terminal α-glucopyranose.	5120	Treatment of LPS-challenged RAW 264.7 macrophages with ALP-1: ↓ TNF-α, IL-6 and IL-1β yet ↑ IL-10; and ↓ LPS-induced NO production in RAW 264.7 macrophages	[54]
Fructan from *Curcuma kwangsiensis*	Plant	β-2,6-linked main chain (81.8% total sugar residues) consisting of single β-fructofuranose branch points (4.9% branching) with both terminal glucose (3.1%) and terminal fructose (5.3%).	5300	↑ phagocytic activity of RAW264.7 macrophages; and ↑ RAW264.7 macrophage proliferation.	[57]
Fructans from *agave salmiana*		N.d, however, agave fructans have been described as highly branched fructose polymers comprising both β-2,6 and β-2,1 linkages [112].	n.d	↑ T-cell-associated transcription factors FOXP3 and Tbet in human PBMCs-showed prebiotic effects	[113]

Abbreviations: ↑, induced or increased; ↓, decreased; DFA-IV, disaccharide Di-D-fructose−2,6′:6,2′-dianhydride (a levan derivative disaccharide consisting of two fructose residues enzymatically produced using levan fructosyl transferase [106]); EPS, exopolysaccharide; FOXP3, forkhead box P3; HPSEC-MALLS, high-performance size-exclusion chromatography coupled with online multiangle laser light scattering; Ig, immunoglobulin; IL, interleukin; iNOS, nitric oxide synthase; n.d, not described; NO, nitric oxide; PBMCs, peripheral blood mononuclear cells; Tbet, T-box transcription factor., TLR, toll-like receptor; TNF, tumour necrosis factor.

**Table 2:** Examples of studies describing the immunomodulatory effects of β-2,6 fructans in vivo.

Fructan Origin	Source	Reported Structure	Mw (Da)	Immunomodulatory Effect(s)	Species	Reference
*Lactobacillus. reuteri* 100-23 levan EPS	Microbial	β-2,6 main chain with β-2,1 branching and terminal fructose	5700 - 7700	↑ splenic CD4+ FOXP3+ regulatory T cells	Rats	[60]
*Bacillus subtilis* natto (fermented soybean) levan EPS	Microbial	n.d.	n.d.	Oral administration ↓ OVA-specific serum IgE levels in mice post-OVA immunisation; ↓ IL-4 levels in splenic T cells.	Mice	[10]
*Bacillus megaterium* 1 (soil) levan EPS	Microbial	n.d	n.d	↑ white blood cell counts, and serum globulin, total protein and lysosome activity.	Fish	[135]
*Acetobacter xylinum* NCIM 2526 levan EPS	Microbial	β-2,6 fructofuranose backbone with β-2,1 branching (Authors refer to the following reference for structural analysis: [97])	750000	After pathogenic challenge, dietary levan: ↑ TNF-α, IL1β, and IL-12p40 expression in several organs, and ↓ expression of intestinal IL-10.	Fish	[137]
*Acetobacter xylinum* NCIM 2526 levan EPS	Microbial	β-2,6 fructofuranose backbone with β-2,1 branching (as described in [97])	n.d	Dietary supplementation to *A. hydrophila*-challenged *Labeo rohita* fingerlings: ↑ Ig levels and ↑ myeloperoxidase; ↑ survival rates; and ↑ TLR22 and ↑ IFN-γ and ↓ TGF-β expression in liver, gill, kidney and intestine.	Fish	[138]
*Arctium lappa* inulin-type fructan, ALP-1	Plant	Glucopyranose-capped β-2,1 fructofuranose main chain with β-2,6 fructofuranose branch points.	5120	Dietary ALP-1: ↑ IL-10 in serum and colon isolates of DSS-induced mice; also ↓ in TNF-α, IL1β and IL-6; and ↑ IgA response in colon; and ameliorated DSS-induced colitis.	Mice	[131]
*Arctium lappa* inulin-type fructan, ALP-1	Plant	Glucopyranose-capped β-2,1 inulin-type fructan containing β-2,6 branch points (see [131])	5120	↓ serum TNF-α, IL-1β and IL-6; and↑IL-10 in LSP-challenged mice; andmodulation of faecal microbiota andSCFA content was observed.	Mice	[54]
*Zymomonas mobilis* levan EPS	Microbial	Described as a microbial levan	700,000	Dietary administration to pigs challenged with LPS or saline (control) via intraperitoneal injection: ↑ blood leukocytes and ↓ IL-6 and TNF-α production in the serum; and Dietary administration also increased Lactobacillus levels in faeces.	Pigs	[139]
*Bacillus* sp. (bacterial honey isolates) levan EPS		Levan comprising β-2,6 fructose-linkages	21,000	Dietary administration promoted gastric ulcer alleviation and ↓ NF-κb production in the gastric mucosa.	Rats	[132]

Abbreviations: ↑, induced or increased; ↓, decreased; DFA-IV, disaccharide Di-D-fructose−2,6′:6,2′-dianhydride (a levan derivative disaccharide consisting of 2 fructose residues enzymatically produced using levan fructosyl transferase [106]); EPS, exopolysaccharide; FOXP3, forkhead box P3; Ig, immunoglobulin; IL, interleukin; n.d, not described; NO, nitric oxide; OVA, ovalbumin; PBMCs, peripheral blood mononuclear cell; TLR, toll-like receptor; TNF, tumour necrosis factor.

## Abbreviations

ATR-FTIR	attenuated total reflectance Fourier-transform infrared spectroscopy;
CD4	cluster of differentiation 4;
CLRs	C-type lectin receptors;
DFA-IV	disaccharide Di-D-fructose-2,6′:6,2′-dianhydride;
DP	degree of polymerisation;
DSS	dextran sodium sulfate;
ELISA	enzyme-linked immunosorbent assay;
EPS	exopolysaccharide;
FITC	fluorescein isothiocyanate;
FOS	fructooligosaccharide;
FOXP3	forkhead box P3;
FT-IR	Fourier transform infrared spectroscopy;
FTF	fructosyltransferase;
GALT	Gut-associated lymphoid tissue;
GC-MS	gas chromatography-mass spectrometry;
GPC	gel permeation chromatography;
GI	gastrointestinal;
HPSEC-MALLS	High-Performance Size-Exclusion Chromatography coupled with on-line multi-angle Laser Light Scattering;
Ig	immunoglobulin;
IL	interleukin;
iNOS	nitric oxide synthase;
KO	knockout;
LPS	lipopolysaccharide;
MAMPs	microbe-associated molecular patterns;
NDP	non-digestible polysaccharides;
NMR	nuclear magnetic reasonance;
NO	nitric oxide;
OVA	ovalbumin;
OVCAR-3	human-derived ovarian carcinoma cells;
MTT	3-(4,5-dimethylthiazol-2-yl)-2,5-diphenyl-2H-tetrazolium bromide;
PBMC	peripheral blood mononuclear cell(s);
PRR	pathogen recognition receptor;
PS	polysaccharides(s)
RT-qPCR	reverse transcription quantitative polymerase chain reaction;
SDS	sodium dodecyl sulfate;
Tbet	T-box transcription factor;
TEER	transepithelial electrical resistance;
TLR	toll-like receptor(s);
TNF	tumour necrosis factor;
WBC	white blood cell(s);
WT	wild type.
